# Suppression of B function strongly supports the modified ABCE model in *Tricyrtis* sp. (Liliaceae)

**DOI:** 10.1038/srep24549

**Published:** 2016-04-15

**Authors:** Masahiro Otani, Ahmad Sharifi, Shosei Kubota, Kanako Oizumi, Fumi Uetake, Masayo Hirai, Yoichiro Hoshino, Akira Kanno, Masaru Nakano

**Affiliations:** 1 Faculty of Agriculture, Niigata University, 2-8050 Ikarashi, Nishi-ku, Niigata 950-2181, Japan; 2 Faculty of Ornamental Plant Biotechnology Research Department, Iranian Academic Center for Education, Culture & Research, Khorasan Razavi Branch, P.O.Box 91775-1163, Mashhad, Iran; 3 Graduate School of Life Sciences, Tohoku University, Katahira 2-1-1, Aoba-ku, Sendai, 980-8577, Japan; 4 Graduate School of Arts and Sciences, The University of Tokyo, Meguro, Tokyo, Japan; 5 College of Bioresource Sciences, Nihon University, Fujisawa, Kanagawa, Japan; 6 Field Science Center for Northern Biosphere, Hokkaido University, Kita 11, Nishi 10, Kita-ku, Sapporo 060-0811, Japan

## Abstract

B class MADS-box genes play important roles in petal and stamen development. Some monocotyledonous species, including liliaceous ones, produce flowers with petaloid tepals in whorls 1 and 2. A modified ABCE model has been proposed to explain the molecular mechanism of development of two-layered petaloid tepals. However, direct evidence for this modified ABCE model has not been reported to date. To clarify the molecular mechanism determining the organ identity of two-layered petaloid tepals, we used chimeric repressor gene-silencing technology (CRES-T) to examine the suppression of B function in the liliaceous ornamental *Tricyrtis* sp. Transgenic plants with suppressed B class genes produced sepaloid tepals in whorls 1 and 2 instead of the petaloid tepals as expected. In addition, the stamens of transgenic plants converted into pistil-like organs with ovule- and stigma-like structures. This report is the first to describe the successful suppression of B function in monocotyledonous species with two-layered petaloid tepals, and the results strongly support the modified ABCE model.

The ABCE model represents the relationship between floral organ development and the expression patterns of three classes of floral homeotic genes, the A, B, and C class genes, most of which encode MADS-box transcription factors^1^,^2^. An A class gene alone specifies sepal formation in whorl 1, the combination of A and B class genes determines petal formation in whorl 2, the combination of B and C class genes specifies stamen formation in whorl 3, and a C class gene alone determines carpel formation in whorl 4. B class genes comprise two paralogous genes, *DEFICIENS* (*DEF*)/*APETALA3* (*AP3*) and *GLOBOSA* (*GLO*)/*PISTILLATA* (*PI*); DEF/AP3 and GLO/PI proteins interact directly and form functional complexes^3^,^4^. *DEF*/*AP3* and *GLO*/*PI* resulted from a duplication event that occurred before the emergence of angiosperms^5^,^6^. Multiple *DEF*/*AP3* or *GLO*/*PI* homologs, present in the genomes of some plant species, have shown functional division by sub- or neo-functionalization^7^,^8^,^9^,^10^. Recently, an E class gene has been identified as an additional class of MADS-box genes, which are involved in the specification of all four types of floral organs^11^,^12^.

In contrast to higher eudicotyledonous species, such as *Arabidopsis thaliana* and *Antirrhinum majus*, some monocotyledonous species, including liliaceous ones, produce two-layered petaloid tepals in whorls 1 and 2. A modified ABCE model has been proposed to explain the molecular mechanisms of organ development of two-layered petaloid tepals (Supplementary Fig. S1)^13^. In this model, the expression of B class genes extends to whorl 1 in addition to whorls 2 and 3, resulting in the development of two-layered petaloid tepals in whorls 1 and 2. The modified ABCE model has been supported by the expression analysis of B class genes in various plant species with two-layered petaloid tepals^14^,^15^,^16^,^17^,^18^,^19^,^20^,^21^,^22^.

In higher eudicotyledonous species, the mutation of B class genes results in the conversion of petals into sepaloid tepals and stamens into carpeloid organs^1^,^23^,^24^,^25^,^26^; similar phenotypes have been induced by suppressing B function in transgenic plants^27^,^28^,^29^. In the liliaceous species *Tulipa gesneriana*, viridiflora cultivars, which have partially greenish tepals in whorls 1 and 2 and slightly degenerated stamens in whorl 3, show lower expression levels of B class genes than normal cultivars^21^. However, the suppression of B function in monocotyledonous species with two-layered petaloid tepals has not been reported to date; thus, there is no direct evidence supporting the modified ABCE model.

*Tricyrtis* spp. are liliaceous ornamental plants with two-layered petaloid tepals. An efficient and reproducible system of *Agrobacterium*-mediated genetic transformation has been established in *Tricyrtis* sp.^30^,^31^. To clarify the molecular mechanism of two-layered petaloid tepal development, we produced and characterized a transgenic *Tricyrtis* sp. in which B function was suppressed through chimeric repressor gene-silencing technology (CRES-T).

## Results

### Phylogenetic relationship of MADS-box genes from *Tricyrtis* sp

**Figure 1:**
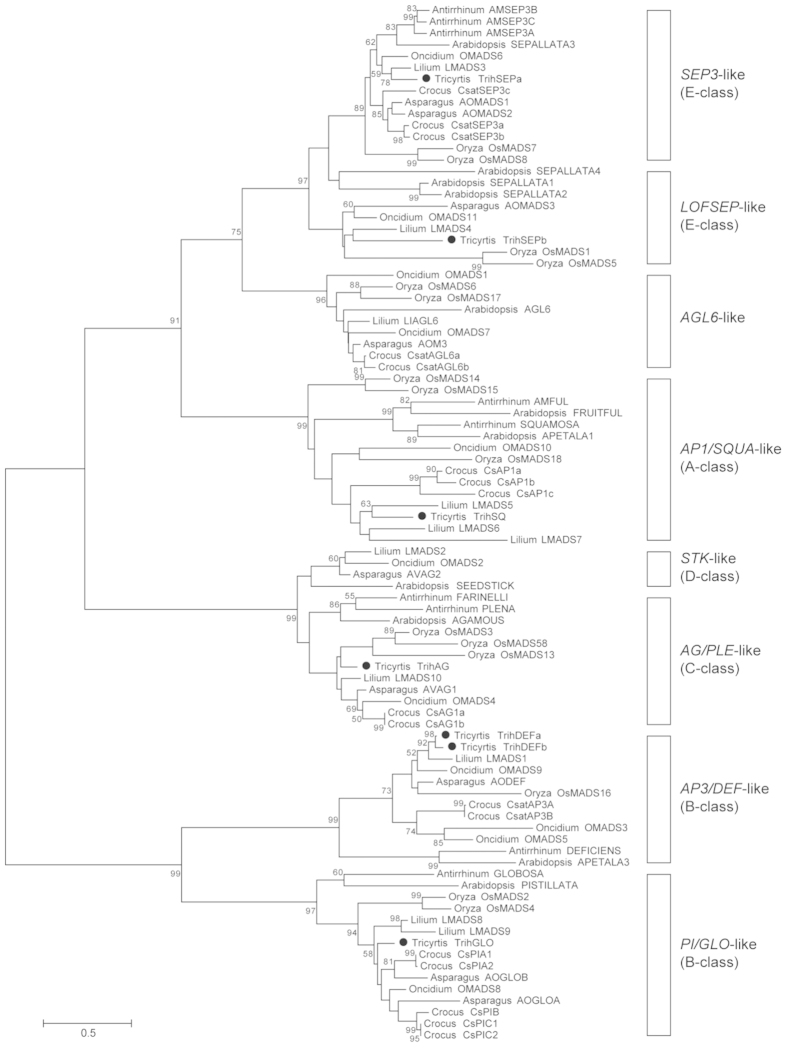
Phylogenetic relationships of MADS-box protein sequences. A phylogenetic tree was constructed using the maximum likelihood method with the JTT + G model. Node values indicate bootstrap support greater than 50% from 1,000 replicates.

Seven MADS-box genes were isolated from *Tricyrtis* sp. Phylogenetic analyses classified these genes into major MADS-box gene lineages: *AP1*/*SQUA*-like (*TrihSQ*), *AP3*/*DEF*-like (*TrihDEFa*, *TrihDEFb*), *PI*/*GLO*-like (*TrihGLO*), *AG*/*PLE*-like (*TrihAG*), and *SEP*-like (*TrihSEPa*, *TrihSEPb*) lineages (Fig. 1). Each *Tricyrtis* sp. gene clustered closely with homologous genes from *Lilium*. The deduced amino acid sequences of *TrihDEFa* and *TrihDEFb* were similar, with only seven differences in amino acid residues. Conversely, the deduced amino acid sequences of *TrihSEPa* and *TrihSEPb* differed greatly; phylogenetic tree analysis assigned *TrihSEPa* to the *SEP3* clade and *TrihSEPb* to the *LOFSEP* clade.

### Expression of floral organ identity genes in *Tricyrtis* sp

**Figure 2:**
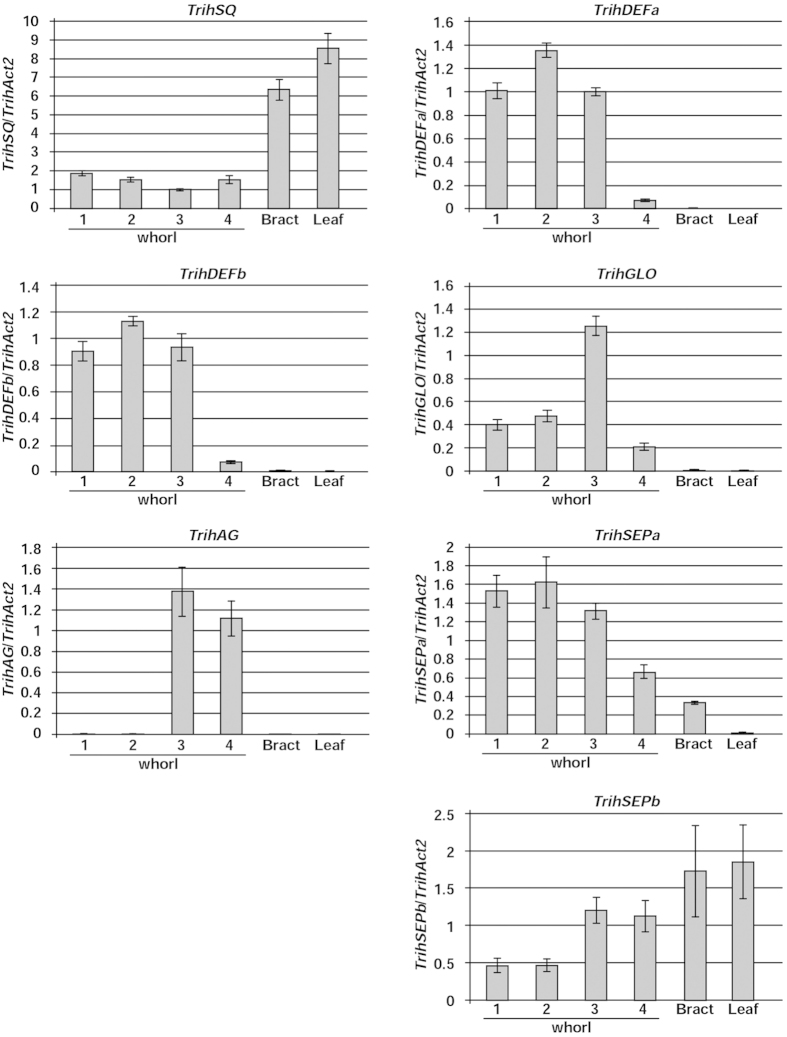
Real-time RT-PCR analysis of endogenous ABCE model gene transcripts in floral organs, bracts, and leaves of wild-type, non-transgenic *Tricyrtis* sp. plants. Relative amounts of transcripts for each gene were normalized to *TrihAct2*. Values represent the means ± standard error of triplicates.

To investigate the spatial expression pattern of floral organ identity genes in a tetraploid variant of *Tricyrtis* sp. ‘Shinonome’, the expression levels of ABCE class genes in the floral organs (i.e., whorl 1, outer tepals; whorl 2, inner tepals; whorl 3, stamens; and whorl 4, carpels), bracts, and leaves of non-transgenic plants were quantified with real-time reverse transcription polymerase chain reaction (RT-PCR) analysis (Fig. 2). The expression of the A class gene *TrihSQ* was detected in all floral organs, bracts, and leaves. Although three B class genes, *TrihDEFa*, *TrihDEFb*, and *TrihGLO*, were expressed in all floral organs, their expression levels were lower in the whorl 4 organs. The C class gene *TrihAG* was strongly expressed in whorls 3 and 4. One E class gene, *TrihSEPa*, was expressed in all floral organs and bracts, whereas the other E class gene, *TrihSEPb*, was strongly expressed in all floral organs and bracts and in the leaves.

### NMorphological characterization of transgenic *Tricyrtis* sp

**Figure 3:**
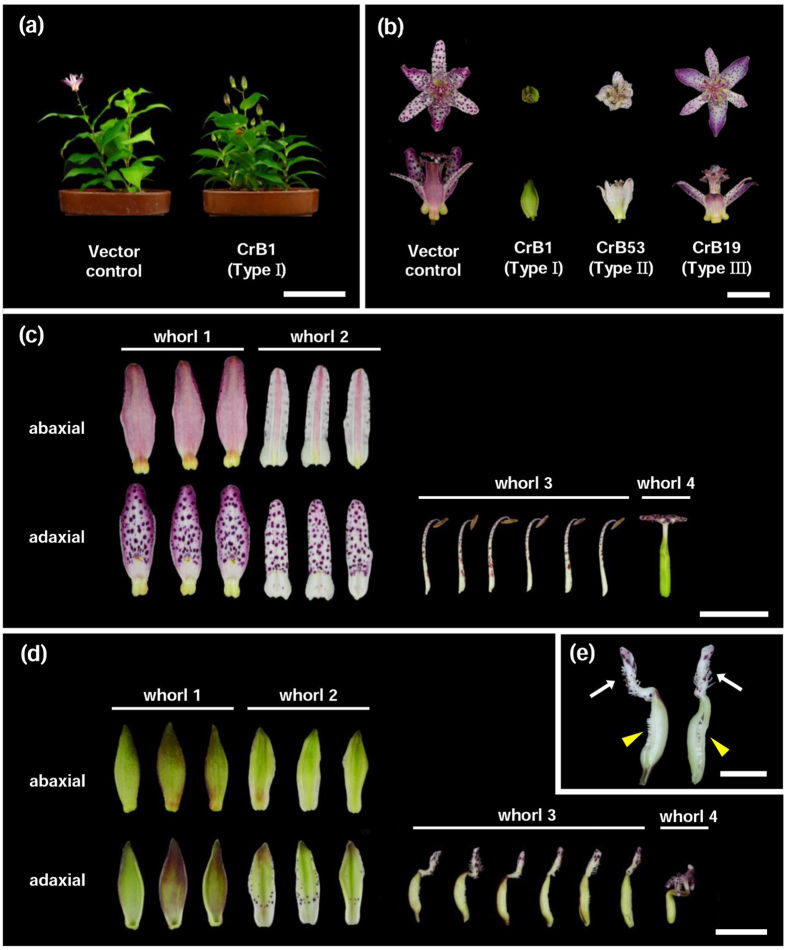
Morphological characterization of transgenic plants containing *TrihDEFa*-*SRDX*. (**a**) Vector control plants and the Type I CrB strain (CrB1) during the flowering season. Bar = 10 cm. (**b**) Flowers in vector control plants and Type I (CrB1), Type II (CrB53), and Type III (CrB19) CrB strains. Bar = 1 cm. (**c**,**d**) Floral organs of (**c**) vector control plants and (**d**) CrB1 plants. Bar = 1 cm. (**e**) Close-up of pistil-like whorl 3 organs of a CrB1 plant. White arrows indicate stigma-like structures. Yellow arrowheads indicate heterotopic ovules. Bar = 5 mm.

To clarify the role of B class genes in the development of two-layered petaloid tepals, transgenic *Tricyrtis* sp. plants, in which B function was suppressed using CRES-T, were produced via *Agrobacterium*-mediated transformation. Forty-one independent transgenic plants carrying an artificial chimeric repressor of *TrihDEFa* (*TrihDEFa*-SRDX; Supplementary Fig. S2) were obtained and termed CrB strains. The presence of the transgene (*HPT*) was confirmed by PCR analysis with the primer set hpt290-F and hpt290-R (Fig. S3). Morphological characterization was performed one year after cultivation in pots during the flowering season. Vector control plants, transformed with *A. tumefaciens* strain EHA101/pIG121Hm^32^, exhibited no phenotypic alterations compared with non-transgenic plants (Fig. 3a–c). CrB strains could be classified into three types according to the degree of phenotypic alteration. Type I CrB strains (CrB1, CrB13, and CrB82) showed significant morphological alterations in floral organs (Fig. 3a,b,d). These strains produced greenish tepals in whorls 1 and 2 instead of petaloid tepals and pistil-like organs in whorl 3 instead of stamens (Fig. 3d,e). The pistil-like organs had a stigma-like structure on the apical part and ovary-like structures on the basal to middle parts (Fig. 3d,e). Furthermore, ectopic ovules were formed on the inside of the ovary-like structures (Fig. 3e). Because the ovary-like structures did not fuse with each other, these ectopic ovules were exposed (Fig. 3d,e). The flowers of Type I CrB strains did not open (Fig. 3b). Although whorl 4 organs (pistil) of Type I CrB strains showed no phenotypic alterations, they were strongly flexed, owing to the unopened state of the flowers (Fig. 3c,d). Type II CrB strains (CrB21, CrB32, CrB45, CrB53, and CrB67) showed moderate morphological alterations to the floral organs. They produced partially greenish tepals in whorl 1, and their flowers were slightly opened (Fig. 3b). In contrast to Type I CrB strains, Type II CrB strains showed no morphological alterations in whorl 2 and whorl 3 organs (Fig. S4). Thirty-three strains were classified as Type III CrB strains and showed no morphological alterations in any floral organs (Fig. 3b). The soil-plant analysis development (SPAD) values of whorl 1 and whorl 2 organs and leaves were measured using a chlorophyll meter. Whorl 1 and whorl 2 organs of both Type I and Type II CrB strains showed higher mean SPAD values than those observed for Type III CrB strains and vector control plants (Supplementary Fig. S5).

**Figure 4:**
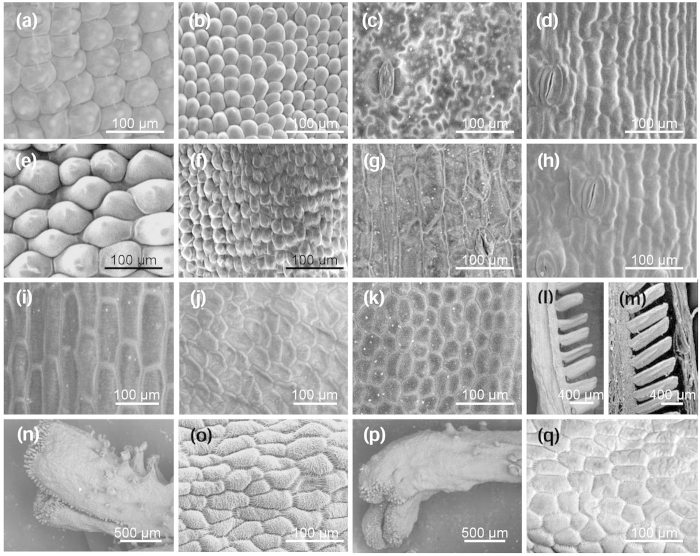
SEM observations of epidermal cells from floral organs of transgenic plants containing *TrihDEFa*-*SRDX*. (**a**,**b**) Adaxial surfaces of whorl 1 organs (outer tepals) of (**a**) vector control plants and (**b**) the Type I CrB strain (CrB1). (**c**,**d**) Abaxial surfaces of whorl 1 organs (outer tepals) of (**c**) vector control and (**d**) CrB1 plants. (**e**,**f**) Adaxial surfaces of whorl 2 organs (inner tepals) of (**e**) vector control and (**f**) CrB1 plants. (**g**,**h**) Abaxial surfaces of whorl 2 organs (inner tepals) of (**g**) vector control and (**h**) CrB1 plants. (**i**) Surface of the middle part of whorl 3 organs (filaments) in vector control plants. (**j**) Surface of the middle part of whorl 3 organs (ovary-like structures) in CrB1 plants. (**k**) Surface of the basal part of whorl 4 organs (ovaries) in vector control plants. (**l**) Ectopic ovules in the middle part of whorl 3 organs (ovary-like structures) in CrB1 plants. (**m**) Longitudinal section of the basal part of whorl 4 organs (ovaries) in vector control plants. (**n**,**o**) Surface of the apical part of whorl 4 organs (stigmas) in vector control plants. (**p**,**q**) Surface of the apical part of whorl 3 organs (stigma-like structures) in CrB1 plants.

The surface of each floral organ of the vector control and Type I CrB strain CrB1 plants was observed through scanning electron microscopy (SEM). In the greenish areas of whorl 1 and whorl 2 organs in CrB1, dome-shaped cells were observed on the adaxial surface; these cells were also observed on petaloid tepals in vector control plants, although the cell size was smaller in CrB1 than in vector control plants (Fig. 4a,b,e,f). The abaxial surface of whorl 1 organs in vector control plants mainly consisted of flat and complex irregular-shaped cells, whereas that in CrB1 plants mainly consisted of slightly swelled and more simple-shaped cells (Fig. 4c,d). The abaxial epidermis of whorl 2 organs in vector control plants mainly consisted of flat cells, whereas that in CrB1 plants mainly consisted of slightly swelled cells (Fig. 4g,h). The surface of the middle part of whorl 3 organs in vector control plants (filaments) contained only rectangular cells (Fig. 4i), whereas CrB1 plants (ovary-like structures) had many irregular-shaped cells that were similar to the ovaries in vector control plants (Fig. 4j,k). Ectopically formed ovules in the middle part of whorl 3 organs in CrB1 showed a structure similar to that of the ovules in vector control plants (Fig. 4l,m). The surfaces of the apical part of the whorl 3 organs in CrB1 (stigma-like structures) had typical papillae (Fig. 4n,p) and were morphologically similar to the stigmas in vector control plants (Fig. 4n–q).

### Expression analysis of endogenous B class genes in transgenic *Tricyrtis* sp

**Figure 5:**
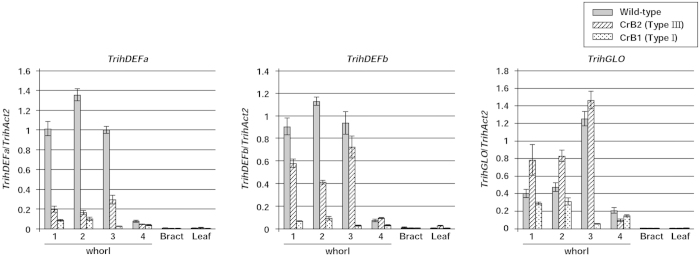
Real-time RT-PCR analysis of endogenous B class MADS-box genes (*TrihDEFa*, *TrihDEFb,* and *TrihGLO*) in floral organs, bracts, and leaves of transgenic plants containing *TrihDEFa*-*SRDX*. Type I (CrB1) and Type III (CrB2) CrB strains showed significant or no morphological alterations, respectively, in floral organs. Relative amounts of transcripts of each gene were normalized to *TrihAct2*. Values represent the means ± standard error of triplicates.

Figure 5 shows the expression levels of endogenous B class genes in floral organs, bracts, and leaves of Type I (CrB1) and Type III (CrB2) CrB strains and in non-transgenic plants. Although the expression of all three endogenous B class genes (*TrihDEFa*, *TrihDEFb*, and *TrihGLO*) was detected in whorls 1, 2, and 3 in CrB1 and CrB2, their expression levels were significantly lower than those observed in non-transgenic plants. The relative amounts of *TrihDEFa* transcript in organs of whorls 1, 2 and 3 in CrB1 decreased to 8.3%, 7.5%, and 2.5% of non-transgenic plant levels, respectively, whereas those in CrB2 decreased to 19.3%, 12.1%, and 29.4% of the levels observed in non-transgenic plants, respectively. The relative amounts of the *TrihDEFb* transcript in organs of whorls 1, 2, and 3 in CrB1 decreased to 7.4%, 8.2%, and 3.1% of non-transgenic plant levels, respectively, and those in CrB2 decreased to 63.9%, 36.5%, and 77.2% of the levels seen in non-transgenic plants, respectively. The relative amounts of *TrihGLO* transcript in organs of whorls 1, 2, and 3 in CrB1 plants decreased to 72.3%, 66.1%, and 4.4% of the levels observed in non-transgenic plants, respectively. In contrast, no apparent differences in the relative amounts of *TrihGLO* transcript in the organs of whorls 1, 2, and 3 were observed between CrB2 and non-transgenic plants. These results indicate that the expression levels of endogenous B class genes in CrB strains are correlated with the degree of morphological alteration.

## Discussion

ABCE model-related MADS-box genes were isolated from *Tricyrtis* sp., and their expression levels were analyzed in non-transgenic plants. Of the B class genes, two *AP3*/*DEF*-like genes (*TrihDEFa* and *TrihDEFb*) and one *PI*/*GLO*-like gene (*TrihGLO*) were isolated. Phylogenetic tree analyses indicated that the duplication of *DEF*-like genes in *Tricyrtis* sp. occurred recently, after the evolutionary split between *Lilium* and *Tricyrtis. TrihDEFa*, *TrihDEFb*, and *TrihGLO* were strongly expressed in whorls 1, 2 and 3 (Fig. 2; Supplementary Fig. S6), indicating that the modified ABCE model^7^ is applicable to *Tricyrtis* sp. The A class gene (*TrihSQ*) was expressed not only in whorls 1 and 2 but also in whorls 3 and 4, bracts and leaves (Fig. 2; Supplementary Fig. S6). Similar extended expression patterns of A class genes have been reported in monocotyledonous species^33^.

In the *Arabidopsis ap3* mutant, petals and stamens are transformed to sepals and carpels, respectively^23^. Transgenic tomato (*Solanum lycopersicum*) plants, in which the B class gene *SlGLO1* is repressed by RNA interference (RNAi), develop greenish petals in whorl 2 and greenish stamens in whorl 3, and the expression of chlorophyll biosynthetic genes is significantly up-regulated in these organs^34^. In the present study, Type I CrB strains produced greenish tepals in whorls 1 and 2 instead of petaloid tepals, and pistil-like organs with ovule- and stigma-like structures were produced in whorl 3 instead of stamens (Figs 3 and 4). Abaxial epidermal cells of the organs of whorls 1 and 2 showed different shapes between Type I CrB strains and the vector control plants (Fig. 4c,d,g,h). In addition, chlorophyll accumulation was observed in organs of whorls 1 and 2 of Type I CrB strains (Supplementary Fig. S5). Thus, there were apparent changes in floral organ identity in whorls 1 and 2 in Type I CrB strains. Because *Tricyrtis* sp. plants do not intrinsically develop sepals, it is difficult to clarify the identity of whorl 1 and whorl 2 organs in Type I CrB strains. In *T. gesneriana*, no apparent morphological differences in the surface structures of whorls 1 and 2 organs were observed between viridiflora cultivars and normal cultivars, although the expression of B class genes in whorls 1 and 2 organs of viridiflora cultivars is lower than in normal cultivars^21^. Thus, greenish tepals caused by the mutation of B class genes might not always exhibit phenotypic alterations in epidermal cells. However, the conversion of stamens into pistil-like organs in whorl 3 of Type I CrB strains, which is one of the typical phenotypes of B class gene mutants such as the *Arabidopsis ap3* mutant^23^, strongly supports that whorl 1 and 2 organs of Type I CrB strains were converted into sepaloid organs. In Type I CrB strains, the flowers did not open (Fig. 3), and the cell sizes of the adaxial surface of whorl 1 and 2 organs were smaller than in the vector control plants (Fig. 4a,b,e,f). Because flower opening is mainly caused by cell expansion at the adaxial side of tepals^35^, unopened flowers of Type I CrB strains might result from the insufficient expansion of epidermal cells in whorl 1 and 2 organs.

B class genes have a positive autoregulatory feedback system that is important for the maintenance of high expression levels^36^. Although there were no differences in the relative amounts of endogenous A, C and E class genes (*TrihSQ*, *TrihAG*, *TrihSEPa* and *TrihSEPb*) transcripts between CrB1 and non-transgenic plants (Supplementary Fig. S6), the expression levels of endogenous B class genes in CrB strains decreased compared with those observed in non-transgenic plants, which might be due to the inhibition of the autoregulatory feedback system. In CrB strains, the expression levels of endogenous B class genes correlated with the degree of morphological alteration in floral organs (Fig. 5), indicating that morphological alterations may have resulted from the suppression of the function of endogenous B class genes. In *A. majus*, the threshold expression level of B class genes required to specify petal identity was approximately 11% of that in the wild-type^37^. The expression levels of endogenous *TrihDEFa* and *TrihDEFb* in organs of whorls 1 and 2 in Type I CrB strains decreased to 7.5–8.3% and 7.4–8.2% of those in non-transgenic plants, respectively (Fig. 5). Thus, the phenotypic alterations observed in organs of whorls 1 and 2 in Type I CrB strains might be caused by a transition in the identity from petaloid to sepaloid organs. Although indirect evidence for the modified ABCE model has been obtained by the expression analysis of B class genes in various plant species with two-layered petaloid tepals^14^,^15^,^16^,^17^,^18^,^19^,^20^,^21^,^22^, the results obtained in the present study directly support the modified ABCE model via the suppression of B class gene function in *Tricyrtis* sp.

## Methods

### Plant materials and growth conditions

Potted plants of *Tricyrtis* sp. ‘Shinonome’ and a tetraploid variant of this cultivar were cultivated in a greenhouse without heating. Tepal-derived embryogenic calli of a tetraploid variant were induced as previously described^38^ and used for transformation. This tetraploid variant showed high transformation efficiency and vigorous growth of transgenic plants compared with the original diploid cultivar ‘Shinonome’.

### Isolation of MADS-box genes

Total RNA was extracted from young flower buds (5–7 mm in length) with an RNeasy Plant Mini Kit (QIAGEN Sciences, Maryland, USA) in accordance with the manufacturer’s instructions. Poly(A)+ RNA was separated from total RNA using DYNABEADS (DYNAL, Oslo, Norway), and cDNA was synthesized using AMV reverse transcriptase (Roche Diagnostics GmbH, Mannheim, Germany). Partial cDNAs were isolated by 3′-rapid amplification of cDNA ends (RACE)^39^ using a 5′/3′-RACE Kit (Roche) and four MADS-box degenerate primers: P038, P041, AD, and SP3^14^. Upstream sequences overlapping the 3′ fragments were isolated by 5′-RACE using a 5′/3′-RACE Kit. cDNA clones with complete open reading frames (ORFs) were isolated via PCR using primers located in the 5′- and 3′-UTR regions with cDNA pools as templates. DNA sequencing was performed using a BigDye Terminator Cycle Sequencing Premix Kit (Applied Biosystems, Foster City, CA, USA) with an automated sequencer (Model 310, Applied Biosystems) according to the manufacturer’s protocol.

### Phylogenetic analysis

The phylogenetic classification of MADS-box genes from *Tricyrtis* sp. was analyzed using MEGA 6.06 software^40^. Genes from *Arabidopsis* (Brassicaceae), *Antirrhinum* (Plantaginaceae), *Oryza* (Poaceae), *Asparagus* (Asparagaceae), *Crocus* (Iridaceae), *Lilium* (Liliaceae), and *Oncidium* (Orchidaceae) were used for the phylogenetic analysis. Accession numbers for these genes are listed in Supplementary Table S1. A molecular phylogenetic tree of the deduced amino acid sequences was constructed using ClustalW implemented in MEGA. To find the most appropriate evolutionary models, maximum likelihood fits for amino acid substitution models were tested using the program included in MEGA. Based on the Bayesian Information Criterion (BIC) score, we selected the JTT + G model and constructed a maximum-likelihood tree with 1,000 bootstrap replicates.

### RNA isolation and gene expression analysis by real-time RT-PCR

Total RNA was extracted using TRIzol reagent (Life Technologies, Carlsbad, CA, USA) and was then treated with DNase I (Life Technologies, Carlsbad, CA, USA) according to the manufacturer’s instructions. For cDNA synthesis, 500 ng of total RNA was reverse transcribed using a PrimeScript™ RT reagent Kit (Takara, Shiga, Japan) in accordance with the manufacturer’s instructions.

RT-PCR analysis was performed using SYBR® Premix Ex Taq™ II (Takara, Shiga, Japan) on a DNA Engine Opticon System (MJ Research, Waltham, MA, USA) as previously described^31^,^41^. The primer sets used in the analysis are listed in Supplementary Table S2. Each PCR was performed in three replicates under the following conditions: initial denaturation at 95 °C for 30 s; 45 cycles of 5 s at 95 °C and 30 s at 60 °C; and plate reading (detection of fluorescent product). To characterize the PCR products, melting curve analysis was performed by slowly increasing the temperature from 60 to 95 °C, and recording fluorescence data at 0.5 °C intervals^42^. The relative amounts of transgene transcripts were calculated using the comparative cycle threshold method, and results were normalized to the actin gene of *Tricyrtis* sp. (*TrihAct2*; AB196260 in the GenBank/EMBL/DDBJ databases).

### Plasmid construction and production of transgenic *Tricyrtis* sp

The *A. tumefaciens* strain EHA101/pIG-CrB was used for transformation. The full-length coding region of *TrihDEFa* was fused with the ERF-associated amphiphilic repression (EAR) motif repression domain (SRDX)^43^. The T-DNA region of the binary vector pIG-CrB contained *TrihDEFa*-SRDX under the control of the cauliflower mosaic virus (CaMV) 35S promoter, the neomycin phosphotransferase II gene (*NPTII*) under the control of the nopaline synthase (NOS) promoter, and the hygromycin phosphotransferase gene (*HPT*) under the control of the CaMV35S promoter (Supplementary Fig. S2). Co-cultivation of embryogenic calli with *Agrobacterium*, selection of transgenic cells and tissues, and regeneration of transgenic plants were performed as previously described^24^. The presence of *HPT* in transgenic plants was confirmed by PCR analysis with the primer set hpt290-F and hpt290-R (Supplementary Table S2).

Transgenic plants were transplanted to pots and cultivated in a growth chamber as previously described^25^. One year after cultivation, morphological characterization was performed during the flowering season. The mean SPAD value, which expresses the relative amount of chlorophylls and has a high correlation with the chlorophyll concentration, of whorl 1 and whorl 2 organs was measured during the flowering season using a chlorophyll meter (SPAD-502; Fujiwara Scientific Co., Tokyo, Japan) as previously described^44^. SEM observation of the surfaces of floral organs was performed as previously described^45^.

## Additional Information

**How to cite this article**: Otani, M. *et al.* Suppression of B function strongly supports the modified ABCE model in *Tricyrtis* sp. (Liliaceae). *Sci. Rep.* **6**, 24549; doi: 10.1038/srep24549 (2016).

## Supplementary Material

Supplementary Information
