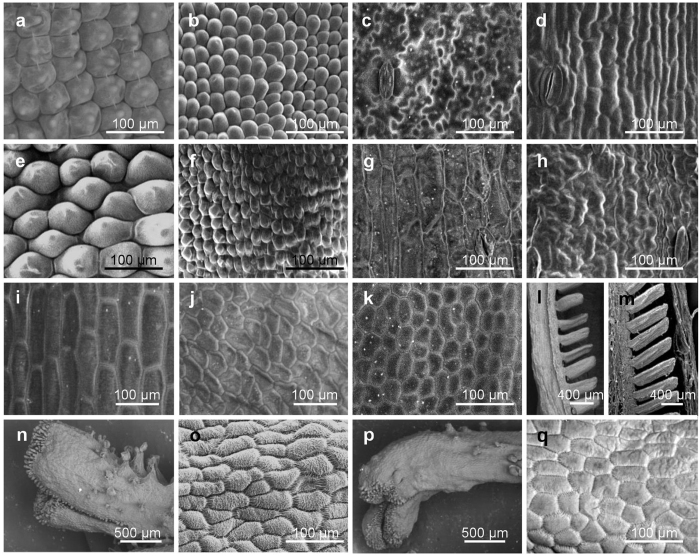# Corrigendum: Suppression of B function strongly supports the modified ABCE model in *Tricyrtis* sp. (Liliaceae)

**DOI:** 10.1038/srep42322

**Published:** 2017-02-09

**Authors:** Masahiro Otani, Ahmad Sharifi, Shosei Kubota, Kanako Oizumi, Fumi Uetake, Masayo Hirai, Yoichiro Hoshino, Akira Kanno, Masaru Nakano

Scientific Reports 6: Article number: 2454910.1038/srep24549; published online: 04 15 2016; updated: 02 09 2017

This Article contains an error in Figure 4, where Figure 4d was inadvertently duplicated in Figure 4h. The correct Figure 4 appears below as Figure 1.

## Figures and Tables

**Figure 1:**