# Supplementary material for: Suppression of B function strongly supports the modified ABCE model in Tricyrtis sp. (Liliaceae)
Source: Sci Rep. 2016 Apr 15;6:24549. doi: 10.1038/srep24549 (PMC4832219; doi:10.1038/srep24549)
Supplement: Supplementary Information [file srep24549-s1.pdf]

Supplementary information

Title of the paper:

Suppression of B function strongly supports the modified ABCE model in *Tricyrtis* sp. (Liliaceae)

Names of the authors:

Masahiro Otani[1†], Ahmad Sharifi[2†], Shosei Kubota[3,4,5], Kanako Oizumi[3], Fumi Uetake[3], Masayo Hirai[3], Yoichiro Hoshino[6], Akira Kanno[3,*], Masaru Nakano[1,*]

Table S1 Accession numbers of genes used in the phylogenetic analysis.

Gene	Species	Accession number
SQUAMOSA	*Antirrhinum majus*	X63701
AMFUL	*Antirrhinum majus*	AY306139
DEFICIENS	*Antirrhinum majus*	AB516402
GLOBOSA	*Antirrhinum majus*	AB516403
PLENA	*Antirrhinum majus*	AB516404
FARINELLI	*Antirrhinum majus*	AB516405
AMSEP3A	*Antirrhinum majus*	AY306140
AMSEP3B	*Antirrhinum majus*	AY306141
AMSEP3C	*Antirrhinum majus*	AY306142
APETALA1	*Arabidopsis thaliana*	Z16421
APETALA3	*Arabidopsis thaliana*	M86357
AGAMOUS	*Arabidopsis thaliana*	X53579
AGL6	*Arabidopsis thaliana*	M55554
FRUITFUL	*Arabidopsis thaliana*	U33473
PISTILLATA	*Arabidopsis thaliana*	D30807
SEEDSTICK	*Arabidopsis thaliana*	U20182
SEPALLATA1	*Arabidopsis thaliana*	M55551
SEPALLATA2	*Arabidopsis thaliana*	M55552
SEPALLATA3	*Arabidopsis thaliana*	AF015552
SEPALLATA4	*Arabidopsis thaliana*	U81369
AODEF	*Asparagus officinalis*	AB180962
AOGLOA	*Asparagus officinalis*	AB103465
AOGLOB	*Asparagus officinalis*	AB103466
AOMADS1	*Asparagus officinalis*	DQ344502
AOMADS2	*Asparagus officinalis*	DQ344503
AOMADS3	*Asparagus officinalis*	DQ344504
AOM3	*Asparagus officinalis*	AY383559
AVAG1	*Asparagus virgatus*	AB125347
AVAG2	*Asparagus virgatus*	AB175825
KCAP1a	*Crocus sativus*	AY337928
KCAP1b	*Crocus sativus*	AY337929
KCAP1c	*Crocus sativus*	AY337930
CsatAP3a	*Crocus sativus*	AY948339
CsatAP3b	*Crocus sativus*	AY948340
CsPIA1	*Crocus sativus*	DQ231247
CsPIA2	*Crocus sativus*	DQ231248
CsPIB	*Crocus sativus*	DQ231249
CsPIC1	*Crocus sativus*	DQ231250

CsPIC2	*Crocus sativus*	DQ231251
CsAG1a	*Crocus sativus*	AY555579
CsAG1b	*Crocus sativus*	AY555580
CsatSEP3a	*Crocus sativus*	EU424137
CsatSEP3b	*Crocus sativus*	EU424138
CsatSEP3c	*Crocus sativus*	EU424139
CsatAGL6a	*Crocus sativus*	EF041505
CsatAGL6b	*Crocus sativus*	EF041506
LlAGL6	*Lilium lancifolium*	GQ496626
LMADS1	*Lilium longiflorum*	AF503913
LMADS2	*Lilium longiflorum*	AY522502
LMADS3	*Lilium longiflorum*	obtained from Tzeng et al. (2003)
LMADS4	*Lilium longiflorum*	obtained from Tzeng et al. (2003)
LMADS5	*Lilium longiflorum*	HQ149331
LMADS6	*Lilium longiflorum*	HQ149332
LMADS7	*Lilium longiflorum*	HQ149333
LMADS8	*Lilium longiflorum*	HQ698550
LMADS9	*Lilium longiflorum*	HQ698551
LMADS10	*Lilium longiflorum*	KJ819937
OMADS1	*Oncidium* Gower Ramsey	HM140843
OMADS2	*Oncidium* Gower Ramsey	KJ819938
OMADS3	*Oncidium* Gower Ramsey	HM140844
OMADS4	*Oncidium* Gower Ramsey	KJ819939
OMADS5	*Oncidium* Gower Ramsey	HM140840
OMADS6	*Oncidium* Gower Ramsey	HM140844
OMADS7	*Oncidium* Gower Ramsey	HM140845
OMADS8	*Oncidium* Gower Ramsey	HM140842
OMADS9	*Oncidium* Gower Ramsey	HM140841
OMADS10	*Oncidium* Gower Ramsey	HM140846
OMADS11	*Oncidium* Gower Ramsey	HM140847
OsMADS1	*Oryza sativa*	L34271
OsMADS2	*Oryza sativa*	L37526
OsMADS3	*Oryza sativa*	L37528
OsMADS4	*Oryza sativa*	AK100233
OsMADS5	*Oryza sativa*	U78890
OsMADS6	*Oryza sativa*	AK069103
OsMADS7	*Oryza sativa*	AK100263
OsMADS8	*Oryza sativa*	U78892
OsMADS13	*Oryza sativa*	AF151693
OsMADS14	*Oryza sativa*	AF058697

OsMADS15	*Oryza sativa*	AF058698
OsMADS16	*Oryza sativa*	AF424549
OsMADS17	*Oryza sativa*	AK069103
OsMADS18	*Oryza sativa*	AF091458
OsMADS58	*Oryza sativa*	AB232157
TrihSQ	*Tricyrtis* sp.	LC080811
TrihDEFa	*Tricyrtis* sp.	LC080806
TrihDEFb	*Tricyrtis* sp.	LC080807
TrihGLO	*Tricyrtis* sp.	LC080808
TrihAG	*Tricyrtis* sp.	LC080805
TrihSEPa	*Tricyrtis* sp.	LC080809
TrihSEPb	*Tricyrtis* sp.	LC080810

Table S2 List of primers used in the present study.

Primer	Sequence (5'→3')	Note
RT-TrihSQ-Fw	TGAGCACTCCTTCCTGATGTCTG	For real-time RT-PCR
RT-TrihSQ-Rev	TTGCCATCTTTAGCCATCCAC	For real-time RT-PCR
RT-TrihDEFa-Fw	CACTTGCTCTAGCGAACGGT	For real-time RT-PCR
RT-TrihDEFa-Rev	TGAAGTAGTTTCACCAAATTCTTTGG	For real-time RT-PCR
RT-TrihDEFb-Fw	TCACTTGTACGAGTTTCGCGTC	For real-time RT-PCR
RT-TrihDEFb-Rev	CATGCATGGATGTCCATAAGG	For real-time RT-PCR
RT-TrihGLO-Fw	TGCTGGAAGAGGAGAACGAAC	For real-time RT-PCR
RT-TrihGLO-Rev	TCCTGTAAATTGGGCTGGATTG	For real-time RT-PCR
RT-TrihAG-Fw	ATGTACCTCCGGAATAAGATAG	For real-time RT-PCR
RT-TrihAG-Rev	GAGAGTAGTGGTGATTGTTGG	For real-time RT-PCR
RT-TrihSEPa-Fw	CAAATTGGGTACCACCCTGATC	For real-time RT-PCR
RT-TrihSEPa-Rev	AGGATTATGAATCAAGTAGTCTGCC	For real-time RT-PCR
RT-TrihSEPb-Fw	ATCAGCCGCCCAGTTCAGAG	For real-time RT-PCR
RT-TrihSEPb-Rev	AAGCAATCCAGGGTGGTCAG	For real-time RT-PCR
SRDX-Rev	TTAAGCGAAACCCAAACGGAG	For real-time RT-PCR
TrihAct2-Fw	TGCCATGTATGTTGCCATTCAG	For real-time RT-PCR
TrihAct2-Rev	AGGGAGTCGGTCAGGTCTCTG	For real-time RT-PCR
hpt290-F	GTGCTTTCAGCTTCGATGTAGG	For detecting *HPT*
hpt290-R	GCTCGTCTGGCTAAGATCGG	For detecting *HPT*

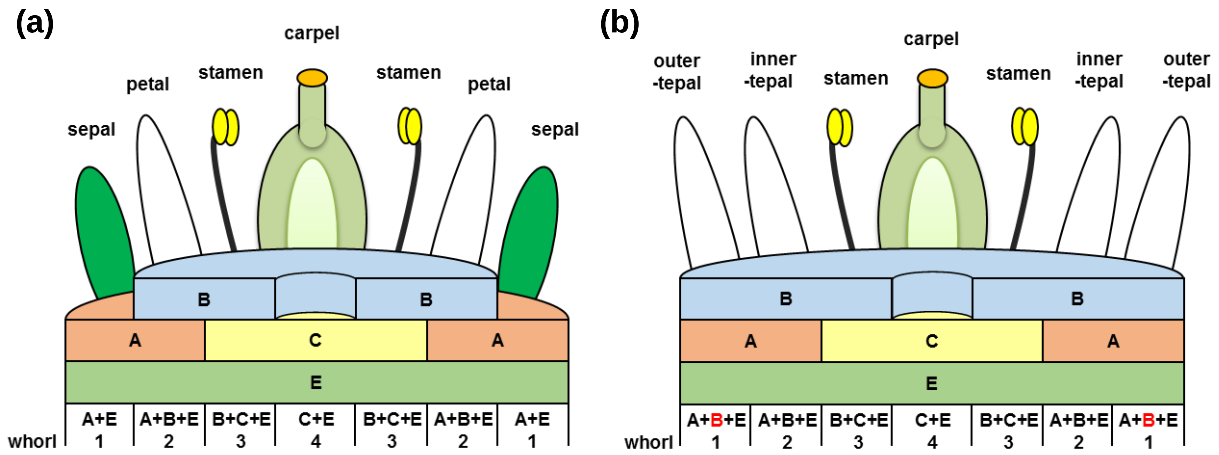

Figure S1 ABCE model (a) and modified ABCE model (b).

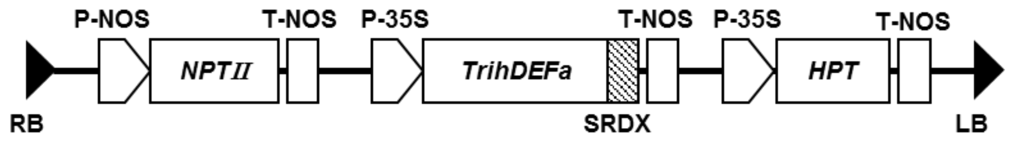

Figure S2 T-DNA region of the binary vector pIG-CrB.

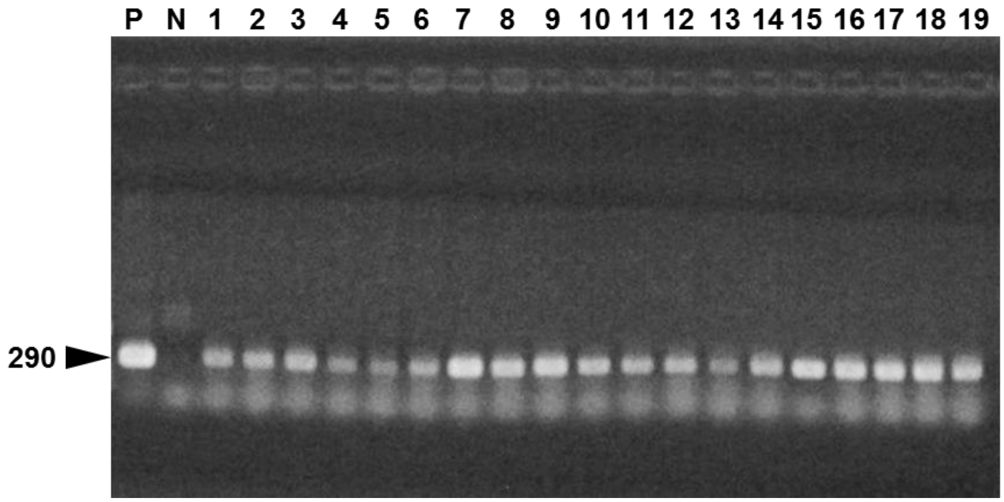

Figure S3 PCR analysis for detecting *HPT* in transgenic plants of *Tricyrtis* sp. Lane P, binary plasmid pIG121Hm as a positive control; Lane N, non-transgenic plant as a negative control; Lanes 1−19, independent CrB strains. Numerals on the left indicate molecular sizes in bp.

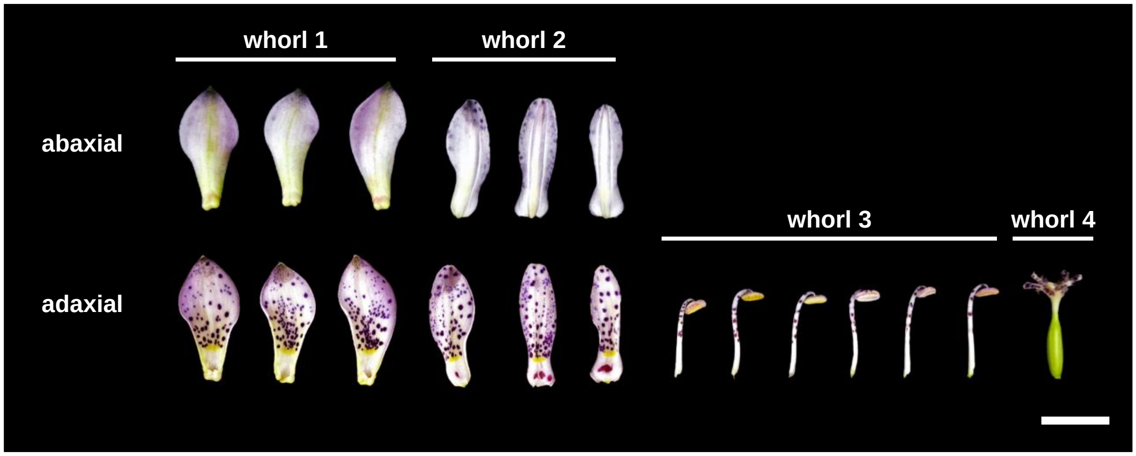

Figure S4 Floral organs of a Type II CrB strain (CrB53). Bar = 1 cm.

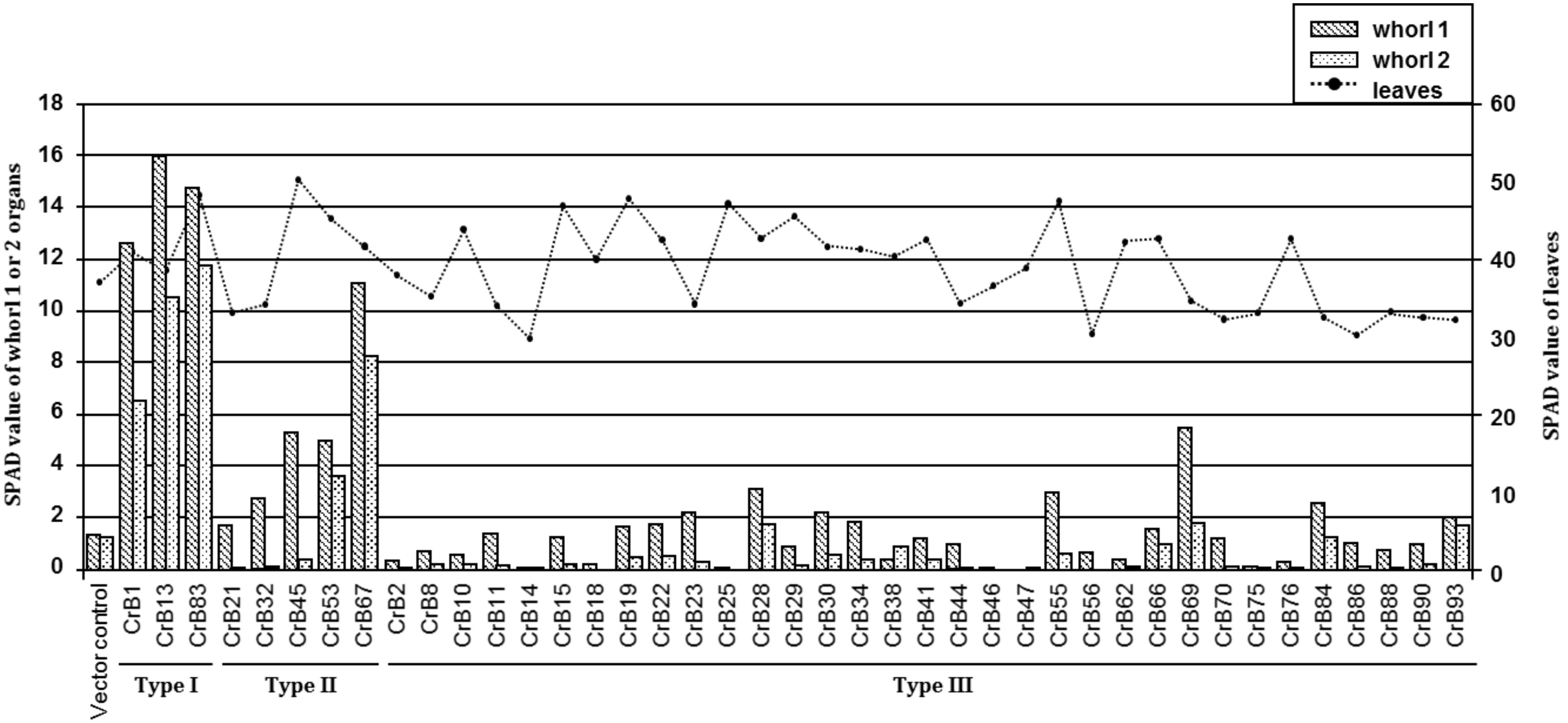

Figure S5 Relative amount of chlorophylls (SPAD value) in whorl 1 and whorl 2 organs, and in leaves of transgenic plants containing *TrihDEFa-SRDX*. Type I, Type II, and Type III CrB strains showed significant, moderate, and no morphological alterations, respectively, in floral organs. Values represent the mean of three organs or leaves for each plant.

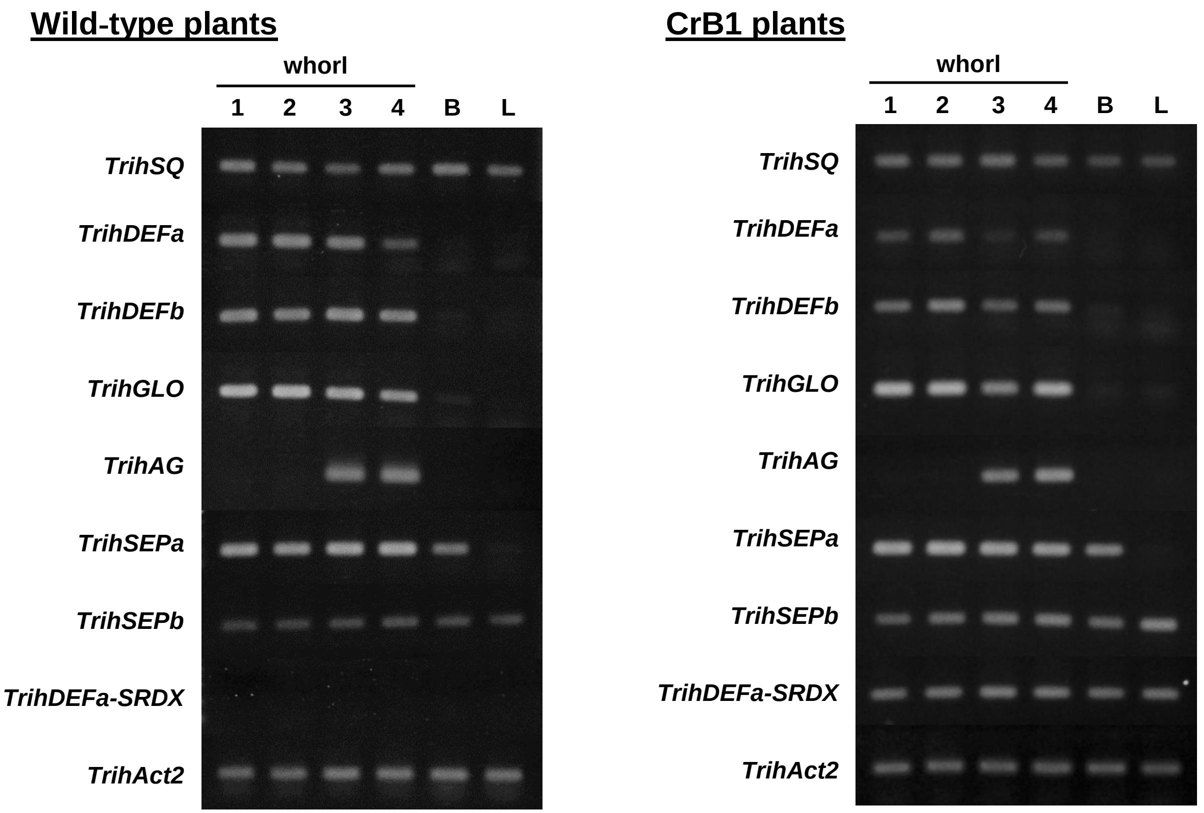

Figure S6 RT-PCR analysis of endogenous ABCE model gene and transgene (*TrihDEFa*-SRDX) transcripts in floral organs, bracts, and leaves of wild-type, non-transgenic *Tricyrtis* sp. plants (left image) and CrB1 plants (right image). B, bracts; L, leaves.
